# Bacterial Exotoxins and the Inflammasome

**DOI:** 10.3389/fimmu.2015.00570

**Published:** 2015-11-10

**Authors:** Allison J. Greaney, Stephen H. Leppla, Mahtab Moayeri

**Affiliations:** ^1^Microbial Pathogenesis Section, Laboratory of Parasitic Diseases, National Institute of Allergy and Infectious Diseases, National Institutes of Health, Bethesda, MD, USA

**Keywords:** inflammasome, caspase-1, interleukin-1, pyroptosis, exotoxins, NLRP3, Nod-like receptors, anthrax lethal toxin

## Abstract

The inflammasomes are intracellular protein complexes that play an important role in innate immune sensing. Activation of inflammasomes leads to activation of caspase-1 and maturation and secretion of the pro-inflammatory cytokines interleukin (IL)-1β and IL-18. In certain myeloid cells, this activation can also lead to an inflammatory cell death (pyroptosis). Inflammasome sensor proteins have evolved to detect a range of microbial ligands and bacterial exotoxins either through direct interaction or by detection of host cell changes elicited by these effectors. Bacterial exotoxins activate the inflammasomes through diverse processes, including direct sensor cleavage, modulation of ion fluxes through plasma membrane pore formation, and perturbation of various host cell functions. In this review, we summarize the findings on some of the bacterial exotoxins that activate the inflammasomes.

## Introduction

Inflammasomes are multiprotein complexes that form in response to microbial effectors, metabolites, nucleic acids, and other danger signals. These signals are sensed by cytosolic pattern recognition receptors [reviewed in Ref. (1)]. The most well-known inflammasome sensors, the nucleotide-binding domain/leucine rich repeat (NLR) proteins, contain common domains, including a leucine rich repeat (LRR), a nucleotide-binding domain (NACHT), a caspase activation and recruitment domain (CARD), and in some, but not all cases, a pyrin domain (PYD). Other inflammasome sensors are the absent in melanoma 2 domain (AIM2) protein, which will not be further discussed in this review, and a sensor called Pyrin, whose function remains controversial and has been described as both an inflammasome activator and inhibitor (2, 3). Upon activation, the inflammasome sensors initiate assembly of a complex that often includes an adaptor protein [usually the apoptosis-associated speck-like protein containing a CARD (ASC)], and pro-caspase-1. Proximity-based autoproteolysis of pro-caspase-1 then leads to cleavage of its substrates – the pro-inflammatory cytokines interleukin (IL)-1β and IL-18, and initiation of a rapid lytic cell death called pyroptosis that requires caspase-1 activity and targeting of unknown death substrates [reviewed in Ref. (1)]. IL-1β and IL-18 are well-studied for their roles in recruiting innate immune cells and promoting adaptive and humoral immunity. Pyroptosis and the accompanying release of cellular contents also act as danger signals, resulting in effects on bystander cells that can impact both innate and adaptive immune responses.

Different sensors activate inflammasome assembly in response to seemingly disparate stimuli. The mechanism of activation of some inflammasome sensors, such as rodent NLRP1, NAIP/NLRC4, and AIM2, are now known [for review see Ref. (1)]. Exactly how the promiscuous NLRP3 inflammasome, however, is activated by a diversity of seemingly disparate stimuli is still a matter of much debate [reviewed in Ref. (1, 4)].

**Figure 1:**
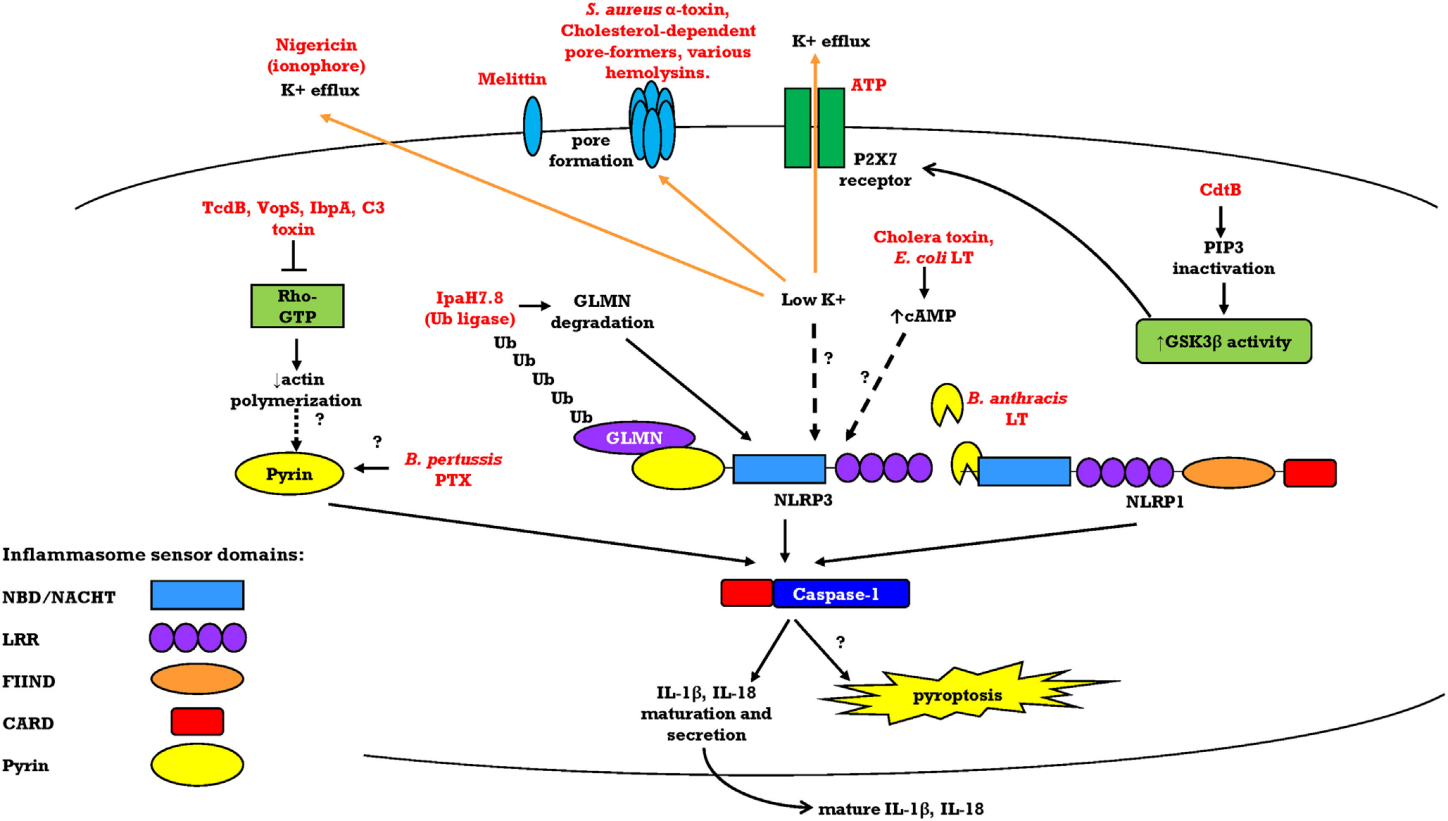
**Overview of mechanisms of toxin-induced activation of the Pyrin, NLRP3, and rodent NLRP1 inflammasomes**. Bacterial exotoxins can activate the inflammasome sensors through diverse direct or indirect mechanisms. After inflammasome sensor activation, these different pathways converge on caspase-1 recruitment to the inflammasome platform and autoproteolytic activation. Caspase-1 cleaves its substrates pro-IL-1β and pro-IL-18 to their mature forms for secretion. Caspase-1 activation is also accompanied by pyroptosis through an unknown mechanism.

This review focuses on effector bacterial exotoxins and how they activate different inflammasome sensors (Table 1; Figure 1). Most toxins that have been described to activate the inflammasomes are pore-forming toxins that activate NLRP3. However, toxins with unique enzymatic actions also activate the inflammasomes. There are also many other bacterial proteins that activate inflammasomes, such as flagellin and needle/rod components of the bacterial type III secretion system (T3SS), which activate NAIP/NLRC4 by direct binding. These are reviewed elsewhere (5–7). Furthermore, the “non-canonical” caspase-11 inflammasome, which directly senses bacterial endotoxin Lipid A, has been examined in a series of elegant studies (8–12) and is also reviewed elsewhere (1, 13, 14). We will discuss bacterial exotoxins that directly activate the inflammasome by sensor modification, namely, *Bacillus anthracis* lethal toxin (LT), and other bacterial exotoxins that activate the NLRP3 and Pyrin inflammasomes indirectly by altering host cell function in ways that include plasma membrane pore formation, co-option of the host actin cytoskeleton, and as-of-yet unknown mechanisms.

**Table 1:** **Inflammasome-activating bacterial exotoxins**.

Toxin	Source	Mechanism	Sensor	Effect sensed by NLR (direct or indirect)	Reference
Lethal toxin (LT)	*B. anthracis*	Protease	NLRP1	NLRP1 cleavage	(19–24)
Nigericin	*S. hygroscopicus*	Ionophore	NLRP3	K^+^ efflux	(40)
α-Hemolysin	*S. aureus*	Pore former	NLRP3	K^+^ efflux	(60–62)
Panton-valentine leukocidin	*S. aureus*	Pore former	NLRP3	K^+^ efflux	(68)
Leukocidin A/B	*S. aureus*	Pore former	NLRP3	K^+^ efflux	(69)
Listeriolysin O	*L. monocytogenes*	Pore former	NLRP3	K^+^ efflux	(42, 43, 50)
Aerolysin	*A. hydrophila*	Pore former	NLRP3	K^+^ efflux	(44)
Tetanolysin O	*C. tetani*	Pore former	NLRP3	K^+^ efflux	(49)
Pneumolysin	*S. pneumonia*	Pore former	NLRP3	K^+^ efflux	(51–53)
β-Hemolysin	Group B *Streptococcus*	Pore former	NLRP3	K^+^ efflux	(55–57)
Streptolysin O	*S. pyogenes*	Pore former	NLRP3	K^+^ efflux	(58, 59)
α-Hemolysin	*E. coli*	Pore former	NLRP3	K^+^ efflux	(77)
Enterohemolysin	*E. coli* O157:H7	Pore former	NLRP3	K^+^ efflux	(76)
Various hemolysins	*Vibrio* species	Pore former	NLRP3	K^+^ efflux	(78–80)
TcdB	*C. difficile*	Glucosylase	Pyrin	Rho GTPase inactivation	(87, 90)
VopS	*V. parahaemolyticus*	Adenylyltransferase	Pyrin	Rho GTPase inactivation	(87)
IbpA	*H. somni*	Adenylyltransferase	Pyrin	Rho GTPase inactivation	(87)
C3 toxin	*C. botulinum*	ADP-ribosyltransferase	Pyrin	Rho GTPase inactivation	(87)
Unknown	*B. cenocepacia*	Deamidase	Pyrin	Rho GTPase inactivation	(87)
Pertussis toxin (PTX)	*B. pertussis*	ADP-ribosyltransferase	Pyrin	Unknown	(87)
CdtB	*Aggregatibacter actinomycetemcomitans*	Lipid phosphatase	NLRP3	GSK3β-induced P2X7 activation	(92, 93)
IpaH7.8	*S. flexneri*	E3 ubiquitin ligase	NLRP3, NLRC4	GLMN degradation	(94)
Pertussis adenylate cyclase toxin	*B. pertussis*	Adenylate cyclase and pore former	NLRP3	Pore formation; K^+^ efflux	(98)
Heat-labile enterotoxin (LT)	*E. coli*	ADP-ribosyltransferase	NLRP3	cAMP increase	(100, 101)
Cholera toxin (CT)	*V. cholerae*	ADP-ribosyltransferase	Unknown	cAMP increase	(101)

## Anthrax Lethal Toxin and Direct Activation of the NLRP1 Inflammasome

Anthrax LT is a major virulence factor of *B. anthracis*, inducing vascular collapse during anthrax infection of animals ranging from rodents to monkeys (15–17). LT is a bipartite toxin made of a receptor-binding moiety, protective antigen (PA), and a zinc-dependent metalloprotease, lethal factor (LF). Upon endosome acidification, PA delivers LF to the host cell cytosol [reviewed in Ref. (18)]. LF activates the NLRP1 inflammasome in macrophages and dendritic cells from certain inbred rodent strains by cleavage of NLRP1 (19, 20). Cleavage occurs in an N-terminal region of unknown function, located in a position corresponding to the PYD of human NLRP1 (19, 21). This cleavage is necessary and sufficient for inflammasome activation (19, 22). In most rat strains, only one of the two NLRP1 paralogs is expressed, and susceptibility to pyroptosis is perfectly correlated with the ability of LT to cleave the expressed NLRP1 sensor (19, 21, 23). Rat strains, such as Fischer and Brown Norway, express NLRP1 variants that are cleaved by LF and have macrophages that pyroptose in response to the toxin. Furthermore, when these rats are challenged with LT, they undergo death in <1 h. Strains such as SHR, Lewis, and Copenhagen have an altered sequence within the LF cleavage site, rendering NLRP1 resistant to cleavage, macrophages resistant to pyroptosis, and the animals highly resistant to toxin challenge (19, 21, 23).

Rat NLRP1 proteins from all tested rat strains are 98% identical (21), pointing to the evolutionary pressure conserving the sensor’s sequence outside of the few polymorphisms in the LF cleavage site. In mice, three *Nlrp1* alleles (*Nlrp1a*, *Nlrp1b*, *Nlrp1c*) exist (24, 25). The one most homologous to the expressed rat NLRP1 is NLRP1a, which is, in a manner similar to rat NLRP1, highly conserved among all mouse strains in which it is expressed1 (25). Murine NLRP1a, however, is not cleaved by LF and its activator is currently unknown.

Rather, the highly polymorphic mouse NLRP1b, which has up to 200 polymorphisms between different inbred mouse strains, is cleaved by the toxin (24, 25). NLRP1 activation in rodent macrophages and dendritic cells and the resulting pyroptosis requires proteasome activity (26–28) and can occur independently of ASC (29–31). Activation results in an inflammatory cytokine (16, 32, 33) and eicosanoid response (34) that confers an increased resistance to *B. anthracis* spore infection (32, 33). Thus, among inbred mouse strains, there is an inverse correlation between sensitivity of macrophages and dendritic cells to LT-induced pyroptosis and animal susceptibility to *B. anthracis* infection.

The unique, rapid death induced by LT in rats, which is not replicated in toxin-sensitive mice at even 10-fold higher doses, does not display the inverse relationship observed in mice. Instead, rat death following both toxin and spore challenge is positively correlated to NLRP1 sensitivity to LT cleavage (35). Human NLRP1 proteins sequenced thus far do not contain an LT cleavage site and instead have an N-terminal PYD. Human NLRP1 is not activated by LT, and human macrophages and dendritic cells are resistant to this toxin (35).

In addition, the obligate intracellular parasite, *Toxoplasma gondii*, has also been shown to activate the NLRP1 inflammasome in select inbred rat strains by an unknown mechanism (36–39). However, it is unknown if *T. gondii* activates NLRP1 in a manner similar to LT, through actions of a protease or toxin. We speculate that NLRP1 has evolved to sense diverse pathogen proteases, and polymorphisms present in different NLRP1 alleles may define responsiveness to different pathogens. Future work is needed to identify other NLRP1 agonists and the pressures driving the evolution of its conserved and polymorphic sequences.

## Pore-Forming Toxins and Indirect NLRP3 Activation

Unlike the toll-like receptors (TLRs) and the NAIP/NLRC4 and AIM2 inflammasomes, which directly sense microbial products, some inflammasome sensors indirectly sense the effects of bacterial toxins on host cell function. For example, NLRP3 is believed to be activated by an indirect mechanism. While the precise signals that activate NLRP3 remain unknown, it has been proposed that NLRP3 may be indirectly activated by K^+^ efflux, lysosomal damage and cathepsin B release, mitochondrial damage, or reactive oxygen species production (1). The best-studied example of indirect inflammasome activation by bacterial toxins is the impact of pore formation on cellular potassium levels and subsequent NLRP3 activation.

Early studies linking IL-1β responses to addition of exogenous ATP or the *Streptomyces hygroscopicus*-derived potassium ionophore, nigericin (40), occurred before inflammasome components were identified and the term introduced. Bhakdi and colleagues confirmed that additional agents that deplete potassium in cells, including the pore-forming *Staphylococcus aureus* alpha toxin, the ionophore valinomycin, and the Na^+^/K^+^ ATPase inhibitor ouabain, can trigger processing of IL-1β (41). These investigators hypothesized that cellular K^+^ concentration changes could control the function of caspase-1. Ten years later, studies with the calcium channel activator maitotoxin, nigericin, and ATP showed that induction of IL-1β and IL-18 secretion in TLR-primed macrophages treated with these K^+^ efflux-inducing agents occurred in a manner dependent on the inflammasome adaptor ASC and the NLRP3 sensor (42). It was found that IL-1β secretion induced by *Listeria monocytogenes* infection of macrophages required listeriolysin expression (42, 43), suggesting that pore formation and perturbation of cellular K^+^ levels could also be the basis for inflammasome activation by this toxin, although the purified toxin itself was not tested.

The first demonstration of NLRP3- and ASC-dependent inflammasome formation in response to a purified pore-forming bacterial toxin was reported with studies using aerolysin purified from *Aeromonas hydrophila* (44). Interestingly, this study identified a novel caspase-1-dependent protective response in cells treated with either aerolysin or *S. aureus* α-hemolysin. In contrast to the pyroptosis usually observed following caspase-1 activation, NLRP3-mediated activation of caspase-1 by sublytic doses of the pore-forming toxins resulted in induction of sterol regulatory element-binding proteins that altered membrane biogenesis and promoted cell survival (44). The link between pore-based lowered intracellular K^+^ and NLRP3 activation was later confirmed by multiple other groups (45, 46). These early studies led to the testing of a large range of purified bacterial lysins and pore formers for their ability to activate the NLRP3 inflammasome.

Other cholesterol-dependent pore-forming toxins similar to aerolysin were also previously linked to IL-1β production (47, 48) and have since been tested in cells with inflammasome component deficiencies to link this response directly to caspase-1 activation. Tetanolysin O, from *Clostridium tetani* induces IL-1β maturation and release from bone marrow-derived macrophages (BMDMs) at low, non-lytic doses in an NLRP3-, cathepsin B-, and caspase-1-dependent manner (49). Listeriolysin, originally suggested to play a role in IL-1β responses during *Listeria* infection via pore formation (42), directly activates the inflammasome by K^+^ efflux induction (50).

*Streptococcus pneumoniae* pneumolysin induces NLRP3-dependent IL-1β secretion that is linked to a pro-inflammatory cytokine cascade, which includes IL-17 and IFN-γ responses (51). Pneumolysin mutants and bacterial serotypes associated with variable toxin production confirm the requirement for this lysin in NLRP3-, ASC- and caspase-1-dependent IL-1β and IL-18 production (52). The host cytokine response to pneumolysin has also been shown to increase protection against pneumonia in a mouse model (52). In a murine model of pneumococcal meningitis, the extent of caspase-1 activation via the NLRP3 inflammasome has been linked to clinical disease severity (53). In contrast to the study described above, in this model, inflammasome activation was associated with pathology rather than bacterial clearance. Pneumolysin was the key inducer of the IL-1β response associated with disease, and inhibitors of IL-1β and IL-18 signaling-altered pathological responses. Pneumolysin activation of the inflammasome was suggested to require ATP release and lysosomal destabilization associated with cathepsin B cytosolic activity (53). Interestingly, pneumolysin has also been linked to the sensing of *S. pneumonia* by the cytosolic DNA-sensing molecule STING and the downstream type I interferon responses, likely in an inflammasome-independent manner (54). Other *Streptococcal* proteins can also mediate inflammasome activation through their pore-forming ability. The pigment associated with group B streptococci was recently shown to be hemolytic and capable of pore formation and induction of a K^+^ efflux linked to NLRP3 activation (55). Previous studies demonstrated that β-hemolysin (56, 57) and other streptococcal lysins (58, 59) also activate the NLRP3 inflammasome.

*Staphylococcus aureus* is another gram-positive bacterium that has evolved to activate the NLRP3 inflammasome through the actions of multiple pore-forming toxins and hemolysins. Following up on Bhakdi’s early observation that *S. aureus* α-hemolysin is associated with the IL-1β response, this toxin was verified to induce NLRP3-dependent activation of caspase-1 in both human and mouse monocytic cells (60). Activation of the inflammasome by α-hemolysin has been reported to have both protective and detrimental consequences *in vivo*. For example, α-hemolysin-mediated activation of NLRP3 in a mouse pneumonia model has been linked to IL-1β-independent necrosis, pulmonary damage, and severe pneumonia (61). By contrast, inflammasome activation is protective in other models. Neutrophil-derived IL-1β in response to α-hemolysin was protective in an abscess model of infection in mice (62). Studies of patients with atopic dermatitis have linked α-hemolysin upregulation of NLRP3 expression and *S. aureus*-mediated caspase-1 activation in monocytes to the control of Th2 responses, which may ameliorate this disease (63). IL-1β has also been linked to protection against *S. aureus*-induced CNS disease and brain abscesses (64). Hemolysin-dependent induction of IL-1β was attenuated in NLRP3- and ASC-deficient microglia following bacterial infection. Interestingly, in these studies, IL-18 secretion from microglia occurred in a manner independent of NLRP3, suggesting a different cleavage mechanism for IL-18 (65). Recently, *S. aureus* phenol soluble modulins (PSMs), another class of pore-forming substances that are important virulence factors [reviewed in Ref. (66)], were shown to induce IL-18 secretion from human keratinocytes in a caspase-1-independent manner (67). In a mouse model, PSMs were also shown to be important for *S. aureus-*induced neutrophil recruitment and systemic inflammation. These authors suggest that IL-18 release and neutrophil recruitment induced by PSMs is inflammasome independent, again suggesting novel mechanisms for IL-18 activation (67).

Other pore-forming toxins from *S. aureus* have also been shown to activate the inflammasome. The Panton–Valentine leukocidin associated with tissue necrosis has been shown to induce release of Il-1β and IL-18 in an NLRP3-dependent manner (68). Leukocidin A/B is another pore-forming toxin that can activate the NLRP3 inflammasome in human monocytes (69). Interestingly, it has also been suggested that *S. aureus* hemolysins do not activate caspase-1 alone and require *S. aureus* lipoproteins for inflammasome activation, albeit independently of the lipoprotein actions on TLR-mediated inflammasome priming (70).

Certain *S. aureus* toxins have also been described to have effects on necroptosis in a manner that may link to their activation of the inflammasome. For example, it was found that the virulent methicillin-resistant (MRSA) strain USA300 induces receptor-interacting serine–threonine kinase (RIP)1/RIP3/mixed lineage kinase domain-like (MLKL)-dependent necroptosis, thereby contributing to inflammatory lung pathology (71). *S. aureus* treatment of human and murine macrophages leads to phosphorylation of MLKL- and necroptosis-induced cell death. *Rip3*^−/−^ macrophages are protected from *S. aureus*-induced cytotoxicity. Live bacteria or supernatants from mutants lacking either the α-hemolysin, leukocidin A/B, or PSM toxins display reduced induction of macrophage cytotoxicity. Interestingly, α-hemolysin-induced inflammasome activation has been shown to be dependent on MLKL pore formation, supporting the link between the NLRP3 inflammasome and necrosome components RIP1, RIP3, and MLKL (71–75). Necroptosis contributes to lung pathology and reduced bacterial clearance *in vivo* by depleting immunoregulatory resident alveolar macrophages that are essential for bacterial clearance (71).

Gram-negative bacteria also produce a variety of hemolysins that have been implicated in activation of the NLRP3 inflammasome. Enterohemorrhagic *Escherichia coli* (EHEC) O157:H7, the causative agent of hemorrhagic uremic syndrome, produces a hemolysin that induces IL-1β from the THP-1 macrophage/monocyte cell line, and RNA-interference experiments indicate a role for NLRP3, ASC, and caspase-1 in the initiation of the cytokine release (76). Uropathogenic *E. coli* also induces IL-1β, and *E. coli* α-hemolysin was found to be responsible for NLRP3-dependent responses in mouse macrophages infected with certain bacterial strains. Other strains activated an NLRP3-independent cell death pathway and hemolysin-independent IL-1β secretion from human macrophages (77). *Vibrio vulnificus* and *Vibrio cholerae* were first shown to activate the NLRP3 inflammasome in macrophages through the action of hemolysins (78). *Vibrio parahaemolyticus* also produces thermostable direct hemolysins known as TDHs that can activate the NLRP3 inflammasome (79). *Vibrio fluvialis*, which induces a diarrheal disease in humans, produces a hemolysin that can activate the inflammasome in mouse and human macrophages. Importantly, in a mouse model, the toxin was associated with IL-1β production, and toxin-containing cell-free culture supernatants induced higher levels of cytokine production. Hemolysin-deficient bacteria and their supernatants had lower levels of response *in vivo* (80).

The study of pore formation-mediated inflammasome activation first started with analysis of marine and fungal ionphores (42) and has now again been extended beyond bacterial toxins. Mold pore-forming mycotoxins (81), viral viraporins (82), melittin, the small cationic pore-forming peptide found in bee venom, which can form a single alpha helix spanning the plasma membrane (83), and the *Bombina maxima* frog derived aerolysin-like protein (84) are examples of unique non-bacterial toxin pore-forming agents that activate the NLRP3 inflammasome and caspase-1. The utility of these and many of the toxins described in this section as potential adjuvants for amplification of the immune response, or pro-inflammatory therapeutics is an area that awaits investigation.

## Indirect Activation of the Inflammasomes by Other Bacterial Exotoxins Having Enzymatic Actions

In addition to NLRP3 activation by pore-forming bacterial toxins, inflammasome activation can also occur by other mechanisms of indirect sensing of bacterial effectors.

Rho family of GTPases is molecular switches that control the dynamics of the actin cytoskeleton. The actin cytoskeleton can be co-opted by bacterial pathogen effectors targeting Rho GTPases for either hyper-activation or inactivation (85). Several cytosolic innate immune-sensing pathways have evolved to recognize the sequelae of Rho GTPase actions. For example, activation of Rho GTPases, including Rac1 and Cdc42, by bacterial toxins activates the cytosolic NOD1 sensor and leads to NFκB-dependent expression of pro-inflammatory genes (86). Recently, Shao and colleagues identified Pyrin as an innate immune sensor of bacterial toxin inhibition of Rho GTPases (87). They demonstrated that the *Clostridium difficile* toxin TcdB, which glucosylates and inactivates Rho proteins, activates the Pyrin inflammasome. This discovery provided a link between the previous findings that Pyrin controlled inflammatory familial Mediterranean fever pathogenesis (88, 89) through inflammasome activation (2), and that *C. difficile* toxins TcdA/B activate the ASC-dependent inflammasome (90). Interestingly, Pyrin specifically recognizes Rho subfamily inhibition, as cytochalasin D, an inhibitor of actin polymerization, and the *Clostridium sordellii* lethal toxin TcsL, which modifies Rac/Cdc42 and some Ras-related GTPases, do not activate the Pyrin inflammasome. Furthermore, a variety of bacterial toxins with different enzymatic activities can activate the Pyrin inflammasome. Other Pyrin activators include *Parahaemolyticus* VopS, *Histophilus somni* IbpA, *Clostridium botulinum* C3 toxin, and *Burkholderia cenocepacia*. These toxins inactivate Rho subfamily GTPases by modifying I-switch residues through glucosylation, adenylation, ADP-ribosylation, and deamidation. Together, the diversity of chemical modifications of Rho GTPases, and the inability to co-IP RHOA/B/C and Pryin suggest that Pyrin does not directly interact with Rho GTPases and detect modifications; rather, it is proposed that Pyrin senses downstream effects on the actin cytoskeleton (87).

Interestingly, the *Bordetella pertussis* toxin PTX, which is an ADP-ribosyltransferase (similar to the botulinum C3 toxin, but targeting a different substrate), has been shown to activate the Pyrin inflammasome *in vivo* (91), as well as to upregulate IL-1β expression in a TLR4-dependent manner. Both events require on the toxin’s ADP-ribosyltransferase activity. While the mechanism of PTX activation of the Pyrin inflammasome is unknown, the intriguing possibility remains that PTX, which ADP-ribosylates the α_i_ subunit of heterotrimeric G proteins, may activate the Pyrin inflammasome via an indirect mechanism similar to that of the other bacterial toxins targeting the GTPases, discussed above.

The *Aggregatibacter actinomycetemcomitans* cytolethal distending toxin (Cdt), can act as both signal 1 and signal 2 for inflammasome activation, up-regulating pro-inflammatory cytokine expression (signal 1) and also activating the NLRP3 sensor (signal 2) (92, 93). Cdt is a heterotrimeric protein found in several bacterial species, also including *Campylobacter jejuni*, *Shigella* species, and some *E. coli* isolates. The CdtA and CdtC subunits comprise the receptor-binding moiety, and the CdtB subunit is an lipid phosphatase. CdtB dephosphorylates the signaling lipid phosphatidylinositol-3,4,5-triphosphate (PIP3) and leads to its degradation, decreased phosphorylation of Akt and glycogen synthase kinase 3β (GSK3β), decreased Akt kinase activity but increased GSK3β kinase activity, and inhibition of the PI-3K signaling pathway. GSK3β activation induced by CdtB in human macrophages and monocytes is believed to lead to NFκB activation and the expression of pro-inflammatory cytokines, including IL-1β, dependent on CtdB lipid phosphatase activity (92). Cdt-induced GSK3β activation leads to the generation of extracellular ATP, activation of the P2X7 purinergic receptor, K^+^ efflux, activation of the NLRP3 inflammasome, and IL-1β release (93). Inflammasome activation is NLRP3, ASC, and caspase-1 dependent (93). This is a case where a single bacterial toxin, once in the macrophage cytosol, may act as both signal 1 and signal 2 for inflammasome activation. Interestingly, it should be noted that Cdt toxins are also considered to act as DNases that induce DNA damage and cause cell cycle arrest. Which of these activities is more important *in vivo* is not clear, and the possibility remains that the DNase activity could also result in activation of other sensors.

The NAIP/NLRC4 inflammasome has been demonstrated to be activated by bacterial flagellin and T3SS needle and rod proteins [reviewed in Ref. (7)]. In contrast to previously known NAIP/NLRC4 agonists, which activate the inflammasome by direct binding to the NAIP sensors, a novel, indirect activator of the NLRC4 inflammasome has recently been identified (94). A *Shigella flexneri* E3 ubiquitin ligase effector protein secreted into the host cell cytosol via the type III secretion system (T3SS), invasion plasmid antigen H7.8 (IpaH7.8), was found to activate the NLRP3 and NLRC4 inflammasomes (94). This protein was found to be an important virulence factor, as enzymatically inactive mutants were defective in lung colonization following intranasal infection. Although in most cases inflammasome activation is important for controlling bacterial infection, this is a case were the activation is used by the bacterium to promote dissemination. The LRR domain of IpaH7.8 was found to directly interact with the host protein glomulin/flagellar-associated protein 68 (GLMN) and target both GLMN and itself for degradation by the proteasome. GLMN was suggested to inhibit the NLRP3 inflammasome through an unknown mechanism. Future studies may reveal whether GLMN inhibits the inflammasome, or if IpaH7.8 activates this sensor through a different mechanism (94).

Interestingly, ricin, a highly poisonous toxin found in the seeds of the *Ricinus communis* plant, and an inhibitor of protein syntheses, has also been shown to induce a macrophage IL-1β-mediated pro-inflammatory response in the airways, contributing to lethality (95). Upregulation of IL-1β transcription and the pro-inflammatory response is believed to be mediated by activation of the stress-activated protein kinases (SAPKs), including JNK and the p38 MAPK [reviewed in Ref. (96)]. Ricin was also demonstrated to activate the NLRP3 inflammasome in a proteasome-dependent manner (97). Inflammasome activation was independent of the ribotoxic stress response and phosphorylation of p38 and JNK. It was proposed that inflammasome activation was the result of breakdown of an unidentified, labile NLRP3 inhibitor whose synthesis is blocked by ricin. JNK- and p38-independent NLRP3 inflammasome activation may contribute to the previous finding that IL-1β plays an important role in ricin-induced severe lung inflammation and lethality (95).

## Bacterial Toxin Inflammasome Activators and Adaptive Immunity

Some studies demonstrated that toxin-induced production of IL-1β also influences adaptive immunity, and it is likely that many toxins that activate the inflammasomes will have similar effects. The pertussis RTX adenylate cyclase toxin previously mentioned contains, in addition to its enzymatic domain, a separate pore-forming domain, which activates the NLRP3 inflammasome. This activation leads to IL-1β production by dendritic cells and induction of antigen-specific Th17 cells that require the cytokine for expansion. Th17 differentiation induced by inflammasome activation is required for control of infection and clearance of bacteria from the lungs in a mouse model (98). Similarly, the IL-17 response associated with NLRP3 inflammasome activation by *S. pneumonia* pneumolysin is also associated with protective immunity against intranasal infection, and IL-1β is required for promoting the IL-17 response (51). Interestingly, the non-toxic trehalose-6,6-dibehenate (TBD) adjuvant, which promotes Th1/Th17 responses, also requires inflammasome adaptor ASC-dependent activation of IL-1β production (99).

The adjuvant role of non-pore-forming bacterial enterotoxins and their enzymatically inactive mutants in inducing inflammasome dependent, Th17-polarized protective immunity has also been studied (100, 101). The *E. coli* heat-labile enterotoxin (HL-LT, commonly termed LT) and cholera toxin (CT) are AB toxins that ADP-ribosylate the Gs component of adenylate cyclase, leading to an increase in cAMP. They have been shown to be potent adjuvants, but their enterotoxicity precludes their use in human oral vaccines. HL-LT along with an enzymatically highly attenuated mutant can both activate the NLRP3/caspase-1 inflammasome and induce mature IL-1β secretion from LPS-primed dendritic cells (100). Inflammasome activation and the associated IL-1β response are required for promoting Th17 responses *in vivo*, which in turn protect against *B. pertussis* challenge in a model where the toxin is the primary adjuvant in the pertussis vaccine (100). In related studies, the heat-killed mycobacteria components in Complete Freund’s Adjuvant (CFA) have been shown to drive Th17 differentiation through a mechanism that requires NLRP3 inflammasome activation (102).

A recent study examined the ability for double-mutant HL-LT and multiple-mutated CT to induce a Th17-mediated adjuvant response in human peripheral blood mononuclear cells (PBMCs) (101). These variants have highly reduced enzymatic activity and, unlike the wild type toxins, are non-toxic but still induce very low levels of cAMP and can stimulate IL-17A production from human PBMCs when administered to cells with a polyclonal T cell superantigen (101). This effect is dependent on cAMP/protein kinase A (PKA) signaling, as a PKA inhibitor inhibits IL-17A production, and a cAMP analog recapitulates the toxin effects. Monocytes were demonstrated to have a modest increase in cAMP in response to the mutant toxins, and monocytes pre-incubated with the toxins and then co-cultured with CD4^+^ T cells led to an increase in IL-17A production in a caspase-1-, IL-1β-dependent manner (101). These studies demonstrate the important role that toxin-induced inflammasome activation may play in vaccine development.

## Concluding Comments

The recognition of self from non-self is the foundation of the innate immune response. Pattern recognition receptors such as the TLRs and some of the inflammasome sensors detect highly conserved microbial molecular patterns, including LPS, peptidoglycan, flagellin, and CpG DNA. The inflammasome receptors discussed in this review, however, are able to detect highly diverse bacterial effectors because they sense these toxins through their functional effects rather than their molecular patterns. In the case of NLRP1, the sensor is activated by direct cleavage by a bacterial protease that also cleaves the mitogen-activated protein kinase kinases (MEKs) and results in cell – and animal – death. The NLRP3 and Pyrin inflammasomes, on the other hand, detect indirect effects on host cell state including ion fluxes and perturbation of the actin cytoskeleton. In this way, the inflammasome sensors have evolved to respond to different bacterial toxins with diverse mechanisms of action to converge on caspase-1 activation and initiation of the immune response.

Toxin activation of the inflammasome in the first-responder cells of the innate immune system plays an important role in pro-inflammatory and pyroptosis events that can have protective or pathogenic consequences in the host. Interestingly, bacterial effectors that inhibit the inflammasome or suppress its activation have also been described in recent years [reviewed in Ref. (103)], suggesting that microbes have also evolved to evade inflammasome detection by the host. With the threat of antimicrobial-resistant bacteria, an understanding of the mechanisms by which microbes modulate the innate immune responses is essential to studies of microbial pathogenesis and therapeutic development.

## Conflict of Interest Statement

The authors declare no commercial or financial relationships that could be construed as a potential conflict of interest.
